# Arbuscular Mycorrhizal Fungi and Molybdenum Act on Different Components of the Cowpea Plant–Soil System Under Salinity

**DOI:** 10.3390/plants15182816

**Published:** 2026-09-14

**Authors:** Murat Tunç, Ferhat Uğurlar, Süreyya Betül Rufaioğlu, Sibel İpekeşen, Levent Yorulmaz, Mihriban Okur, Doğan İpekeşen, Behiye Tuba Biçer

**Affiliations:** 1 Department of Field Crops, Faculty of Agriculture, Harran University, Şanlıurfa 63300, Turkey; murattunc@harran.edu.tr; 2 Department of Soil Science and Plant Nutrition, Faculty of Agriculture, Harran University, Şanlıurfa 63300, Turkey; fugurlar@harran.edu.tr (F.U.); sureyyarufaioglu@harran.edu.tr (S.B.R.); 3 Department of Field Crops, Faculty of Agriculture, Dicle University, Diyarbakır 21280, Turkey; sibel.ipekesen@dicle.edu.tr (S.İ.); levent.yorulmaz@dicle.edu.tr (L.Y.); 4 Department of Agricultural Machinery and Technologies Engineering, Faculty of Agriculture, Dicle University, Diyarbakır 21280, Turkey; mihriban.okur@dicle.edu.tr; 5 Department of Plant Protection, Faculty of Agriculture, Dicle University, Diyarbakır 21280, Turkey; dogan21n@gmail.com

**Keywords:** cowpea (*Vigna unguiculata* L.), arbuscular mycorrhizal fungi, molybdenum, salinity stress, soil enzyme activity, mycorrhizal colonization

## Abstract

This study evaluated the combined effects of arbuscular mycorrhizal fungi (AMF) and two molybdenum (Mo) doses on cowpea (*Vigna unguiculata* L., cv. Karnıkara) grown under saline and non-saline soil conditions. A greenhouse experiment was conducted using a 2 × 6 factorial design with three replications, and morphological, physiological, biochemical and mycorrhizal traits were measured. The Mo doses were deliberately set above the agronomic range in order to test how the symbiosis behaves under Mo excess. Salinity reduced fresh root weight by 53% and root length by 30%, whereas stomatal conductance and soil catalase activity increased by 87% and 379%, respectively. None of the treatments changed plant height, plant fresh weight, stomatal conductance, root length or root fresh weight: the main treatment effect and the treatment × soil interaction were not significant for any plant growth trait. Under saline conditions, AMF applied alone produced the highest mycorrhizal density (3.7%), and the addition of Mo reduced it, indicating a dose-dependent suppression of colonization rather than a synergistic enhancement. The AMF × Mo interaction was significant for soil enzyme activities but not for plant biomass, indicating that the combination acted mainly on the soil biochemical compartment. Overall, AMF and Mo influenced largely separable components of the cowpea plant–soil system under salinity. Under the experimental conditions, AMF application under salinity was associated with more favorable responses in terms of root development and stomatal characteristics, whereas the combined application of supra-optimal Mo doses with AMF did not provide a marked improvement in these responses. These results should be interpreted within the context of the application doses and experimental conditions used in this study. Further field studies encompassing different soil properties, salinity levels, Mo doses, and growing conditions are needed before these findings can be directly generalized to broader agronomic conditions.

## 1. Introduction

Soil salinization is one of the most important environmental constraints on agricultural productivity worldwide, affecting approximately 20% of global agricultural land [1]. The problem is particularly severe in semi-arid regions, where high evaporation and unsustainable irrigation practices promote the accumulation of salt ions in the soil [2]. Salinity stress affects plant–soil–microorganism interactions by reducing soil water potential, disrupting root membrane integrity, and suppressing microbial activity [3]. Osmotic stress and nutrient imbalance further restrict plant growth by decreasing photosynthesis and increasing respiration [4]. Cowpea (*Vigna unguiculata* L.) is a strategic legume and an important component of food security in many regions because of its nutritional value and versatile uses [5]. However, the nitrogen fixation-based metabolism of legumes makes them sensitive to environmental stress [6]. Under saline conditions, increased oxidative stress and suppressed growth can lead to substantial yield losses [3], through processes such as impaired water uptake, ion toxicity, and the accumulation of reactive oxygen species (ROS) [7,8,9]. Sustainable approaches for maintaining plant performance under salinity are therefore of considerable practical interest.

In this context, arbuscular mycorrhizal fungi (AMF) are important biological components that can enhance plant stress tolerance. AMF form symbiotic associations with plant roots and improve the uptake of water and nutrients, particularly phosphorus [10]. Through extensive hyphal networks, AMF allow plants to access resources beyond the root zone and modify root architecture toward a more efficient absorption system [11,12,13]. AMF have also been reported to reduce cellular damage by promoting antioxidant activation, regulating hormonal balance, improving osmotic adjustment, and enhancing antioxidant enzyme activities under salinity [14,15,16,17]. The magnitude of these effects varies with soil properties, salinity level, and fungal strain. AMF applications also influence soil enzyme activities such as phosphatase, dehydrogenase and urease, thereby modifying nutrient cycling under saline conditions [18,19,20].

Molybdenum (Mo) is an additional factor in plant nutrition under saline conditions. As a structural component of enzymes such as nitrate reductase and nitrogenase, Mo has a central role in nitrogen metabolism [21]. Mo can promote plant growth at optimal doses, whereas excessive concentrations may have adverse effects [22]. Previous studies indicate that Mo becomes particularly relevant under salinity stress, where the demand for efficient nitrogen utilization increases [23,24,25]. Mo also contributes to ROS detoxification by enhancing the activities of antioxidant enzymes such as superoxide dismutase and peroxidases [26,27,28]. What happens when Mo and AMF are supplied together is much less clear, and the available evidence points in more than one direction. In legume systems, Mo supplied alongside AMF has been reported to improve nitrogen accumulation and assimilation [6], and combining AMF with nano-scale boron, zinc and Mo increased colonization and metabolite content in stevia [29]. Other work gives the opposite picture. In alfalfa grown along a Mo gradient, the benefit of the symbiosis was dose-dependent and shifted towards a trade-off as Mo supply increased [30], and in maize exposed to toxic Mo levels, AMF acted mainly by protecting leaf performance rather than by raising colonization [21]. Mo therefore behaves as a partner or as a constraint for the symbiosis depending on the dose, the host species and the soil, and no consistent picture of the underlying dose–response has yet emerged. What is still missing is a description of where in the plant–soil system the two factors actually act when they are applied together under salinity: whether they converge on the same responses, or whether one operates mainly through the plant and the other mainly through the soil. Answering that question requires plant, soil-biochemical and mycorrhizal responses to be measured in the same pots, which is the approach taken here. The two Mo concentrations correspond to approximately 163 and 326 mg Mo kg^−1^ soil, respectively, which are well above the rates recommended for legume production. In legumes, Mo is generally applied at rates ranging from tens to a few hundred grams per hectare [24]. These doses were selected not to develop a fertilization recommendation, but rather to evaluate the response of the symbiosis under Mo excess. This approach is consistent with previous studies that used supra-optimal Mo doses to characterize AMF–Mo interactions in maize and alfalfa [21,30]. The present study therefore examined the combined effects of AMF inoculation and two Mo doses (0.3% and 0.6%, *w*/*v*) on cowpea grown under saline and non-saline conditions. In addition to plant growth parameters, stomatal conductance, soil enzyme activities (catalase, urease and dehydrogenase) and mycorrhizal parameters were measured. The aim was to determine whether AMF and Mo act on the same or on different components of the plant–soil system under salinity.

## 2. Results

### 2.1. Descriptive Statistics and Two-Way ANOVA

The soil factor had a highly significant effect on plant weight, stomatal conductance, root length and root weight, while its effect on plant height was not significant. The treatment × soil interaction was not significant for any growth parameter, so the pattern seen for plant growth did not reach significance under the conditions tested; the absence of a significant treatment effect indicates that an effect of AMF, Mo or their combination on plant growth was not detected in this experiment, rather than that these factors have no effect. For soil enzyme activities and chemical properties, both treatment and soil factors had highly significant effects on catalase, urease, dehydrogenase, pH and EC, and the treatment × soil interaction was also significant for all of these parameters (Table S1). For mycorrhizal parameters, which were analyzed across the three AMF-containing treatments, the soil factor was highly significant for all three parameters (Table S2). Treatment affected spore number (*p* < 0.05) and mycorrhizal frequency (*p* < 0.01), and the treatment × soil interaction was highly significant for mycorrhizal frequency and mycorrhizal density. The treatment main effect on mycorrhizal density was not significant on its own (*p* = 0.051), which follows from the interaction: the ranking of the treatments reverses between the two soils, so the two patterns cancel when they are averaged. Because the treatment × soil interaction was significant for all three mycorrhizal parameters, the corresponding treatment and soil main effects should be interpreted in the light of that interaction rather than on their own.

### 2.2. Mycorrhizal Parameters

**Figure 1:**
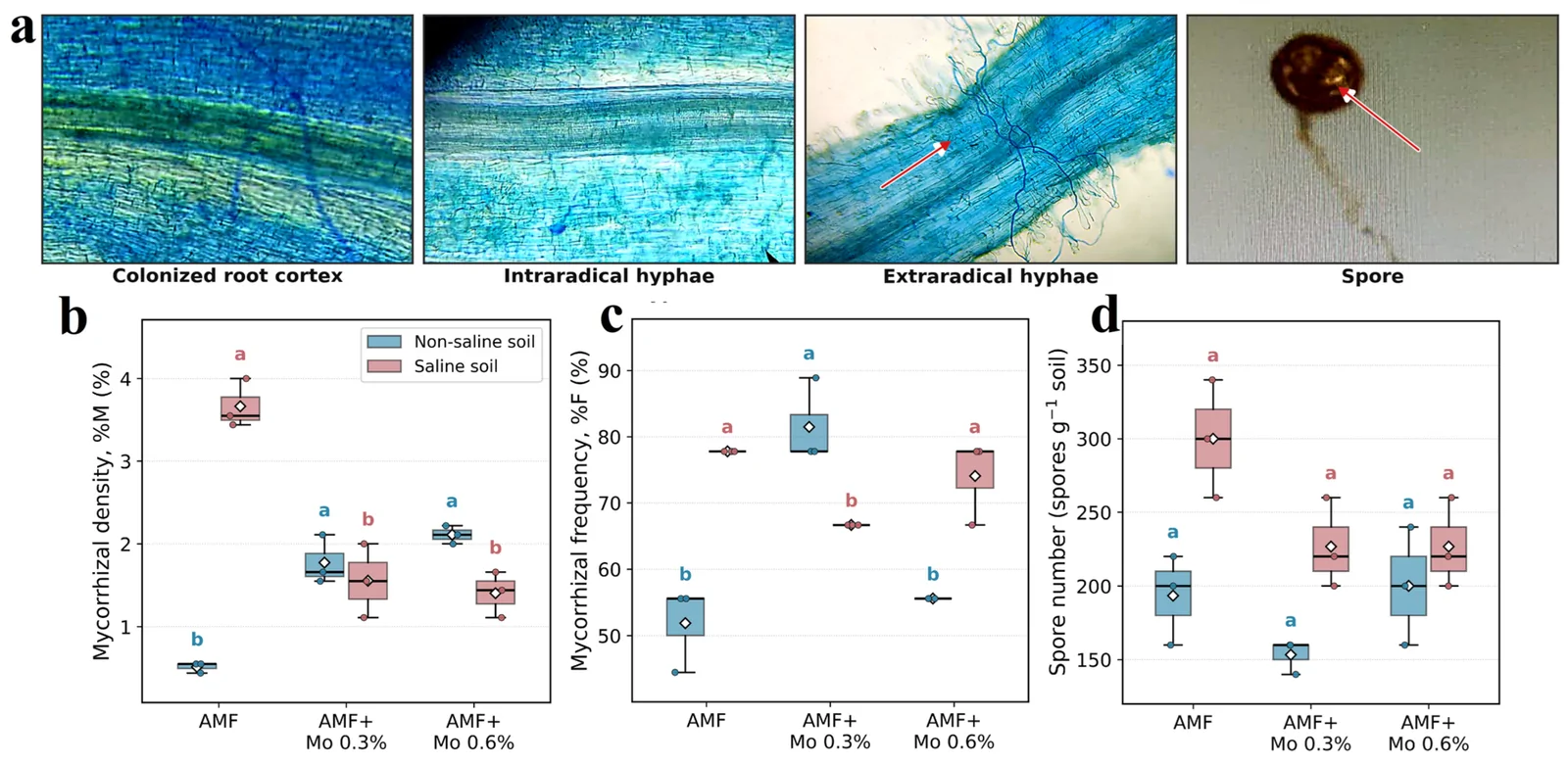
Arbuscular mycorrhizal colonization of cowpea roots and mycorrhizal parameters under saline and non-saline conditions. (**a**) Representative trypan-blue-stained light micrographs showing a colonized root cortex, arbuscules, a vesicle, and intraradical and extraradical hyphae. (**b**) Mycorrhizal density (%M, the proportion of root cortex colonized), (**c**) mycorrhizal frequency (%F, the proportion of root fragments containing fungal structures) and (**d**) spore number (spores g^−1^ soil). Different letters indicate significant differences among the treatment × soil combinations according to Tukey’s HSD test at *p* < 0.05.

Mycorrhizal density (%M) was lowest (0.4–0.6%) under non-saline conditions with AMF applied alone and increased to 1.6–2.2% when Mo was added. Under saline conditions, the highest mycorrhizal density was recorded in the AMF treatment (3.4–4.0%), whereas the addition of Mo reduced it to 1.1–2.0% (Figure 1; Tables S2 and S3). Mycorrhizal frequency (%F) was lowest (44–56%) under non-saline conditions with AMF alone and increased to 56–89% with Mo application. Under saline conditions, frequency varied within a narrower range (67–78%), with the highest values again in the AMF treatment. Spore numbers under non-saline conditions were lowest in the AMF + 0.3% Mo treatment (140–160 spores g^−1^ soil) and highest in the AMF and AMF + 0.6% Mo treatments (160–240 spores g^−1^ soil). Under saline conditions, spore numbers were generally higher (200–340 spores g^−1^ soil), with the highest values in the AMF treatment (Figure 1).

### 2.3. Soil Enzyme Activities and Chemical Properties

**Figure 2:**
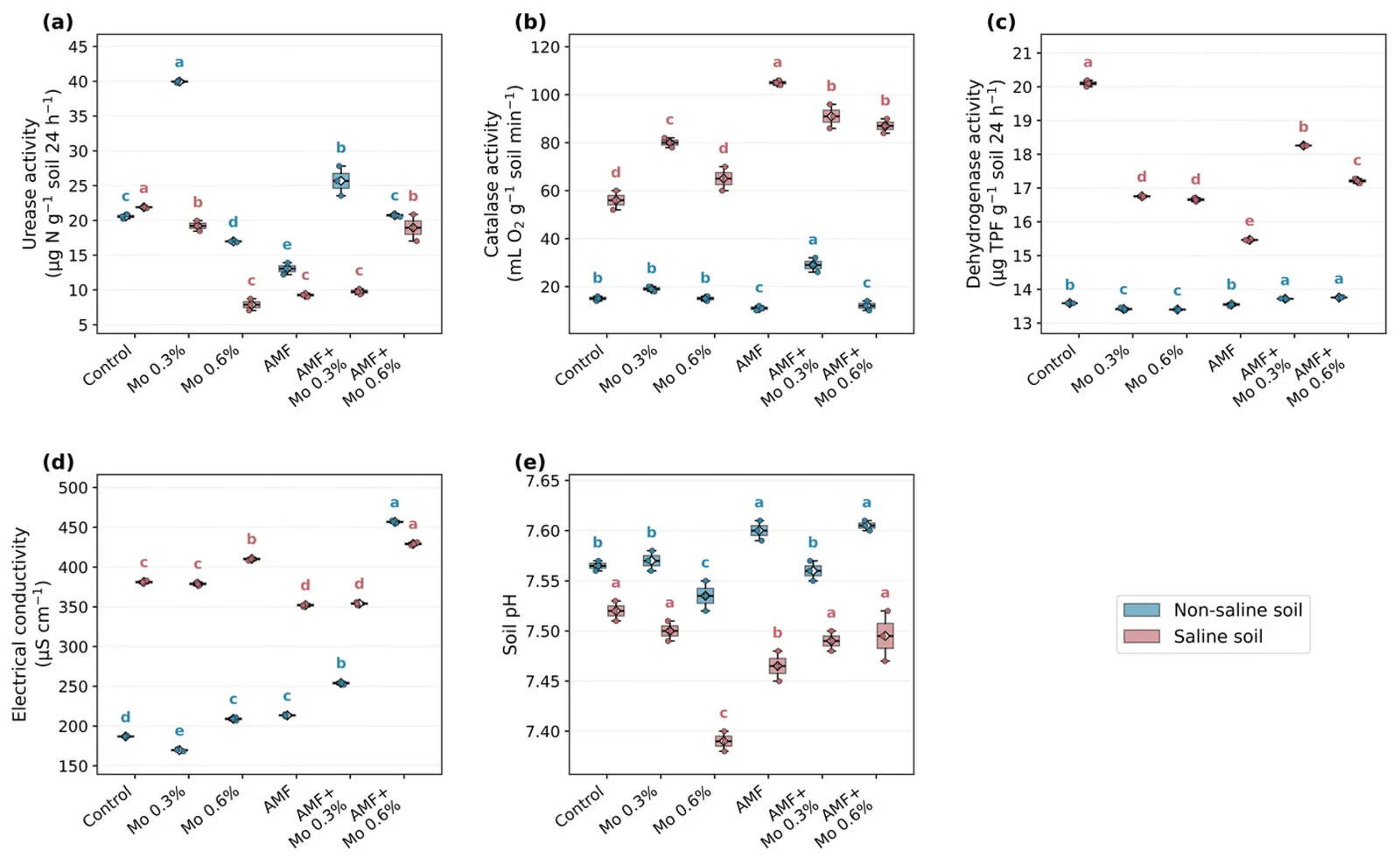
Effects of the treatments on soil enzyme activities and physicochemical properties under non-saline (blue) and saline (pink) conditions: (**a**) urease activity, (**b**) catalase activity, (**c**) dehydrogenase activity, (**d**) electrical conductivity (EC) and (**e**) soil pH. Boxes show the interquartile range, horizontal lines the median, open diamonds the mean and points the individual observations. Different letters indicate significant differences among the treatment × soil combinations according to Tukey’s HSD test at *p* < 0.05.

Urease activity was highest under non-saline conditions in the 0.3% Mo treatment (39.8–40.1 µg N g^−1^ 24 h^−1^) and lowest in the AMF treatment (12.2–13.9 µg N g^−1^ 24 h^−1^). Under saline conditions, urease activity was generally lower (7.0–22.0 µg N g^−1^ 24 h^−1^), with the lowest values in the 0.6% Mo, AMF and AMF + 0.3% Mo treatments (Figure 2; Tables S1 and S3). Catalase activity varied within a narrow range under non-saline conditions (10–32 mL O_2_ g^−1^ min^−1^) but increased markedly under saline conditions to 52–106 mL O_2_ g^−1^ min^−1^, with the highest values in the AMF and AMF + Mo treatments; averaged across treatments, salinity increased catalase activity by 379% (Figure 2). Dehydrogenase activity varied within a narrow range under non-saline conditions (13.4–13.8 µg TPF g^−1^ 24 h^−1^) and increased under saline conditions to 15.4–20.2 µg TPF g^−1^ 24 h^−1^, with the highest values in the control and the lowest in the AMF treatment (Figure 2). EC ranged between 168 and 458 µS cm^−1^ under non-saline conditions, with the upper end of this range corresponding to the AMF + 0.6% Mo treatment, whereas under saline conditions EC values were consistently high (350–431 µS cm^−1^) (Figure 2). Soil pH varied within a narrow range under non-saline conditions (7.52–7.61) and was slightly lower under saline conditions (7.38–7.53).

### 2.4. Plant Growth Parameters

**Figure 3:**
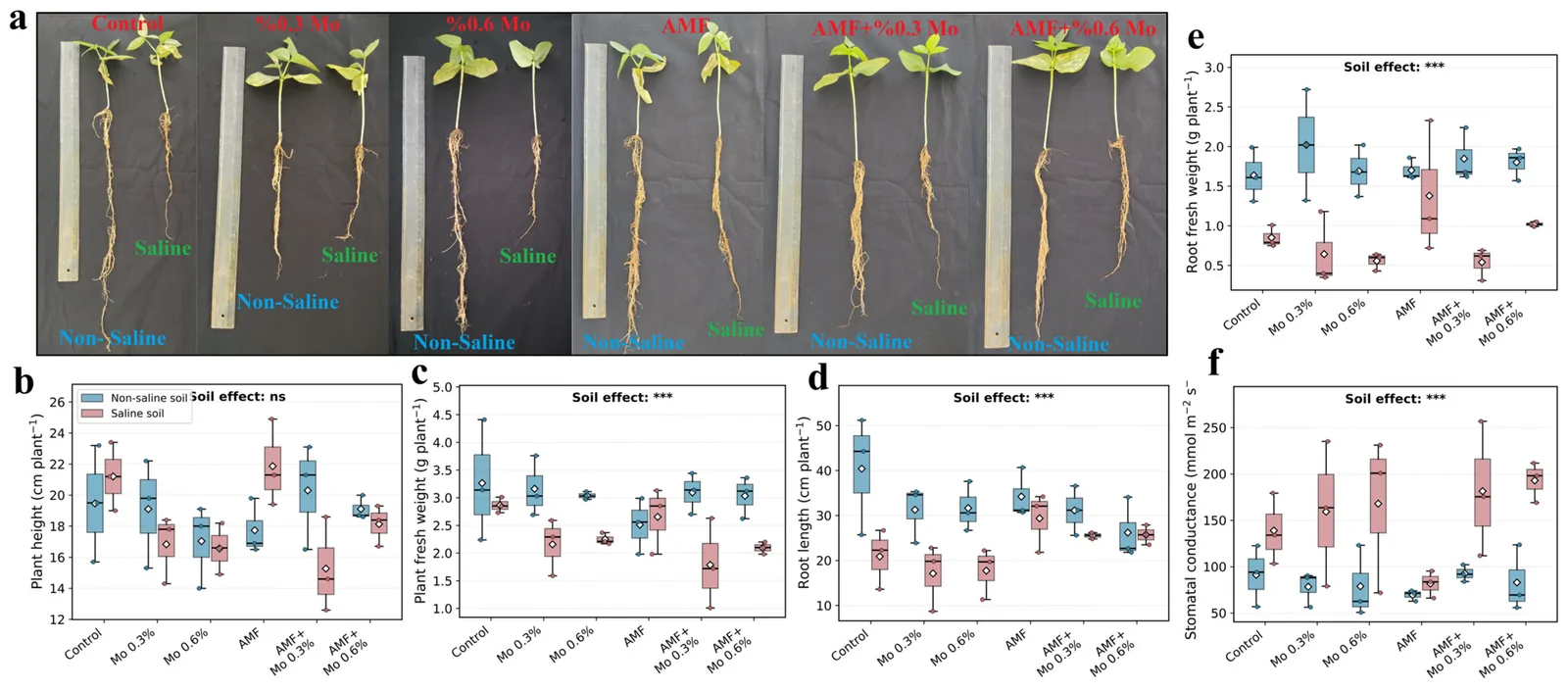
Plant growth and physiological responses of cowpea to the six treatments under non-saline and saline soil. (**a**) Representative cowpea plants for each treatment in non-saline and saline soil (soil condition labelled within each photograph). Box plots of (**b**) plant height, (**c**) plant fresh weight, (**d**) root length, (**e**) root fresh weight and (**f**) stomatal conductance; boxes show the interquartile range, horizontal lines the median, open diamonds the mean and points the individual observations. ns, not significant; *** *p* < 0.001.

Plant height was similar under both soil conditions (13–25 cm), and the difference was not statistically significant (Figure 3; Tables S1 and S3). Plant weight, root length and root weight differed significantly between soil types. Under non-saline conditions, plant weight (2.0–4.4 g plant^−1^), root length (21.8–51.2 cm) and root weight (1.3–2.7 g plant^−1^) were higher, whereas under saline conditions these parameters were reduced to 1.0–3.1 g plant^−1^, 8.7–34.2 cm and 0.3–2.3 g plant^−1^, respectively; averaged across treatments, salinity reduced fresh root weight by 53% and root length by 30%. Stomatal conductance was higher under saline conditions, ranging from 66 to 257 mmol m^−2^ s^−1^ compared with 51–124 mmol m^−2^ s^−1^ under non-saline conditions, corresponding to an average increase of 87%. Within saline pots, AMF applied alone gave lower stomatal conductance (82 mmol m^−2^ s^−1^) than the saline control (139 mmol m^−2^ s^−1^), whereas the AMF + 0.6% Mo combination gave higher values (193 mmol m^−2^ s^−1^).

### 2.5. Principal Component Analysis, Clustering and Correlations

**Figure 4:**
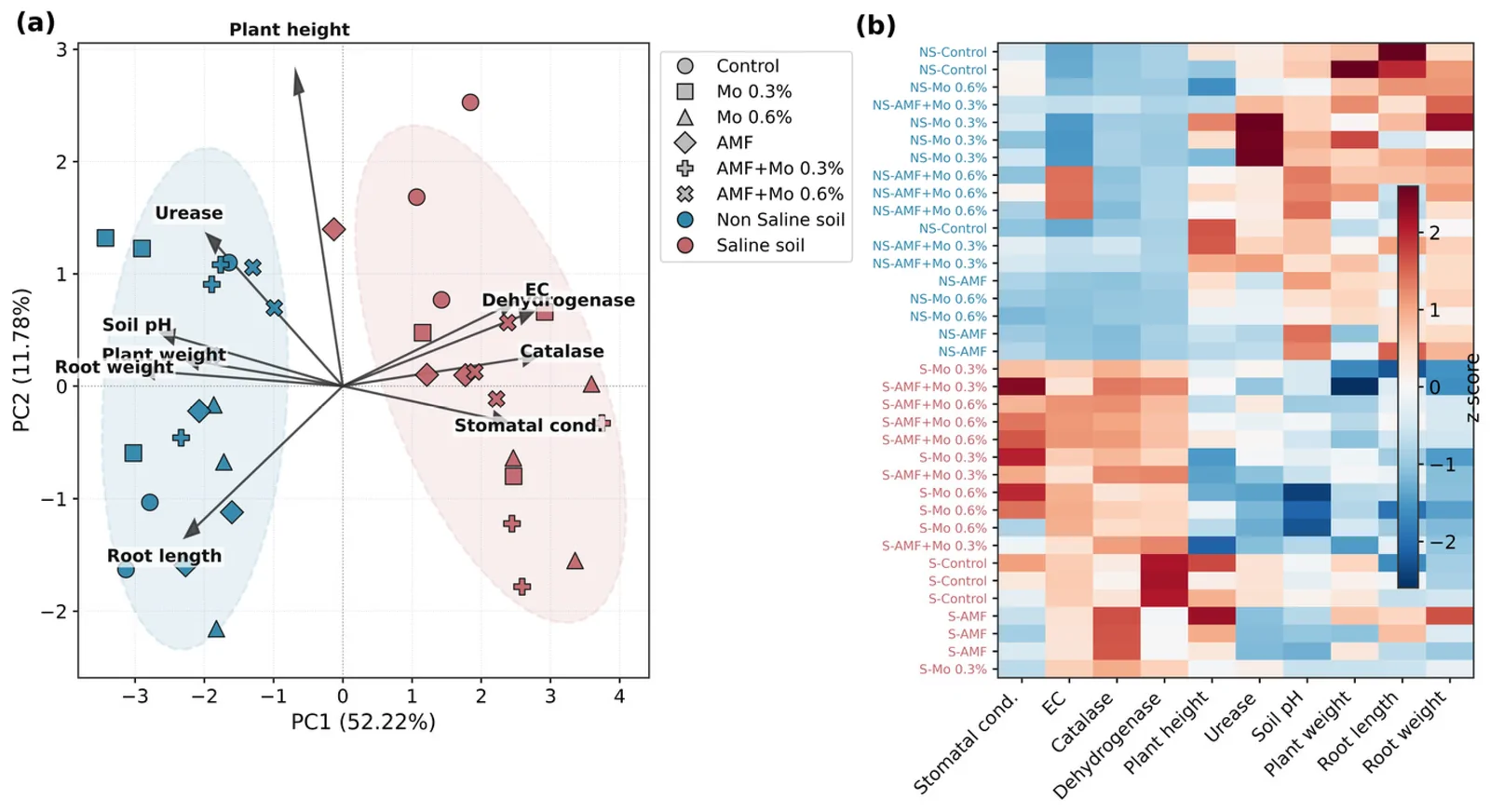
Multivariate analysis of plant and soil responses under saline and non-saline conditions. (**a**) PCA biplot. Ellipses represent the 95% confidence regions of the saline and non-saline groups and vectors indicate the direction and magnitude of variable loadings. (**b**) Hierarchical clustering heatmap (Ward’s method, Euclidean distance).

PCA showed a clear separation between saline and non-saline soil conditions. PC1 explained 52.22% and PC2 11.78% of the total variance (Figure 4a); the analysis was based on the ten variables measured in every pot, and each of the six treatments is shown with its own symbol. Stomatal conductance, dehydrogenase, catalase and EC were positively associated with PC1 and aligned with saline conditions, whereas plant growth parameters (plant height, plant weight, root length, root weight), urease and pH were negatively associated with PC1 and corresponded to non-saline conditions. Within each soil condition the six treatments overlapped extensively along PC1, which is consistent with soil condition rather than treatment being the main source of variation. A second PCA restricted to the AMF-inoculated pots and including the mycorrhizal variables (Figure S1; PC1 42.56%, PC2 20.10%) placed spore number, mycorrhizal frequency and density in intermediate positions between the two groups of variables. Hierarchical clustering supported the PCA pattern: samples grouped into two main clusters by soil type, while variables clustered into functional groups, with enzyme activities and stomatal conductance forming one cluster and growth parameters another (Figure 4b).

**Figure 5:**
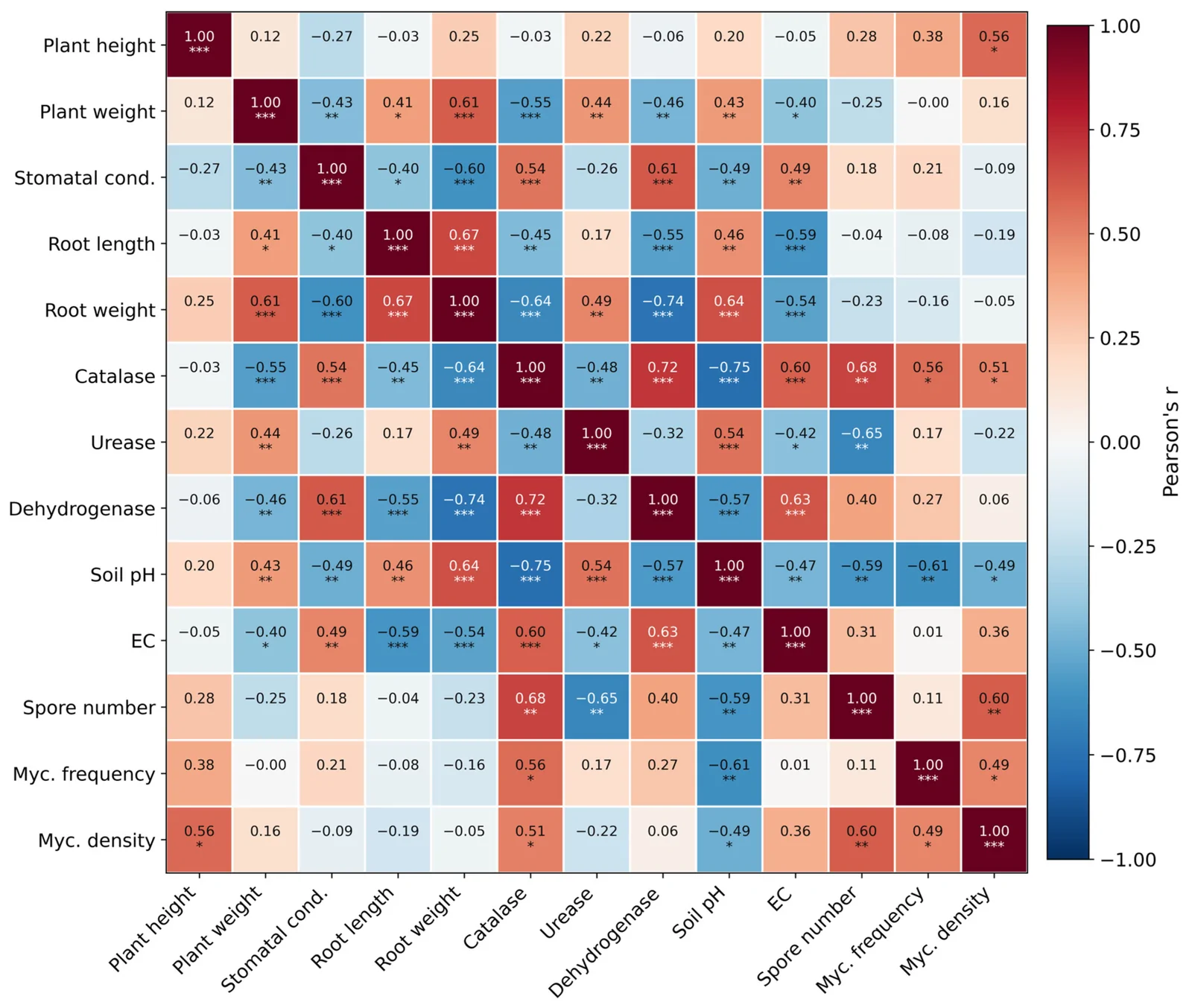
Pearson correlation coefficients among plant growth traits, physiological traits, soil properties and mycorrhizal parameters across the whole dataset. Blue and red cells indicate negative and positive correlations, respectively; asterisks denote significance levels (* *p* < 0.05; ** *p* < 0.01; *** *p* < 0.001).

Pearson correlation analysis across the whole dataset (Figure 5) showed that plant growth parameters were positively inter-correlated (root length–root weight *r* = +0.67, plant weight–root weight *r* = +0.61) and negatively related to catalase activity and EC (root weight–catalase *r* = −0.64; root weight–EC *r* = −0.54), while catalase, dehydrogenase, EC and stomatal conductance formed a second, positively inter-correlated group (catalase–dehydrogenase *r* = +0.72; stomatal conductance–dehydrogenase *r* = +0.61). Urease activity was positively associated with the growth parameters and with soil pH (*r* = +0.54).

### 2.6. Mo Dose–Response and Its Interaction with AMF

**Figure 6:**
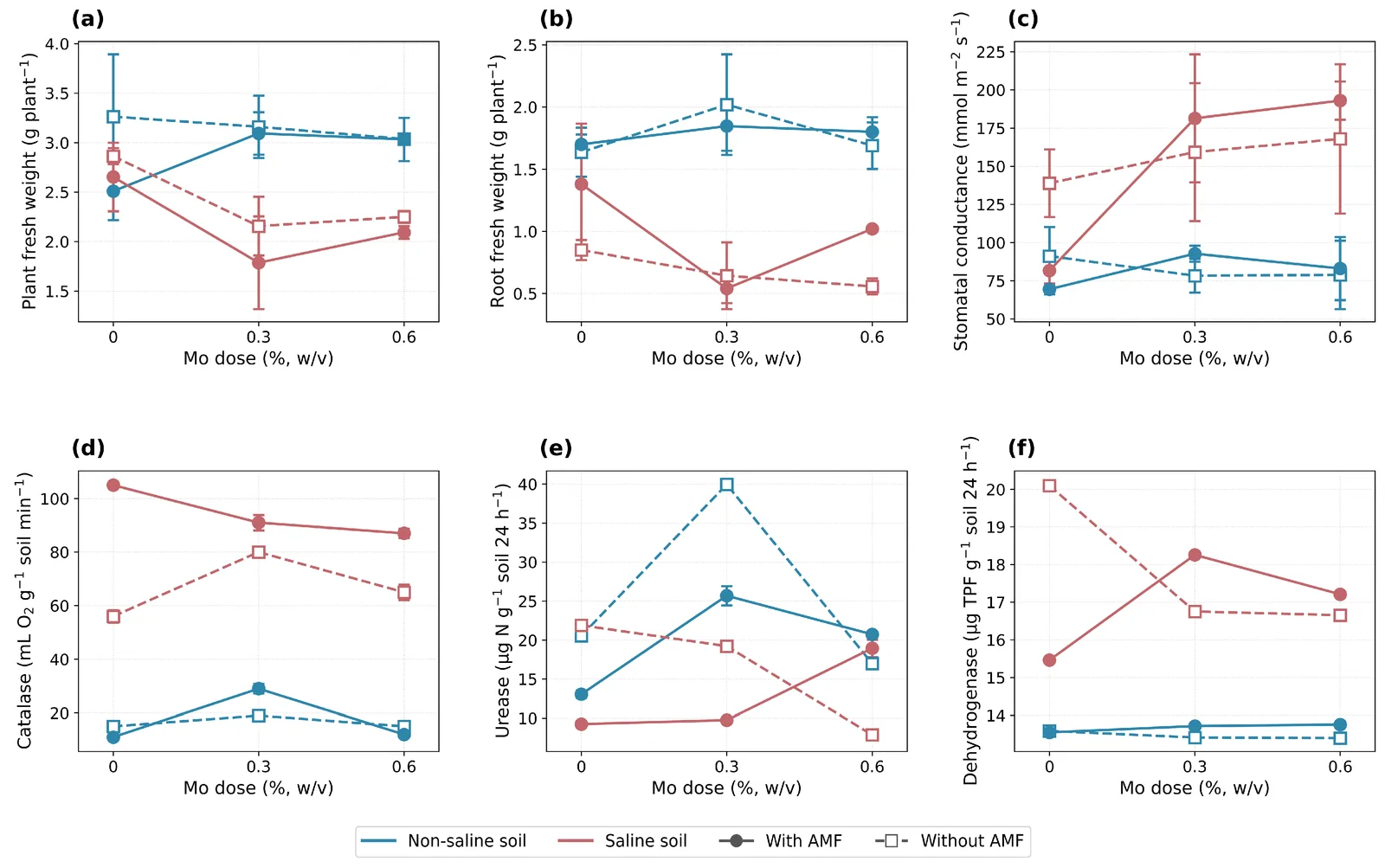
Dose–response of selected parameters to Mo (0, 0.3 and 0.6%, *w*/*v*) under saline and non-saline conditions, in the presence and absence of AMF inoculation. Symbols show treatment means and error bars the standard error of the mean. The AMF × Mo interaction was tested by two-way ANOVA on the saline pots, with Mo dose and AMF status as factors.

The dose–response analysis (Figure 6) separated the two compartments of the system quite clearly. Mo had little influence on plant growth parameters, and the presence of AMF did not change that: for plant weight, root weight and stomatal conductance, the AMF × Mo interaction under salinity was not significant (*p* = 0.92, 0.37 and 0.42, respectively), so the responses to Mo were statistically indistinguishable with and without AMF, even though the AMF and non-AMF series were displaced relative to one another (Figure 6, Table 1). Soil enzyme activities behaved differently.

For soil enzyme activities, the AMF × Mo interaction was highly significant under salinity for catalase (*F* = 43.0, *p* < 0.001), urease (*F* = 284.9, *p* < 0.001) and dehydrogenase (*F* = 5077, *p* < 0.001). Catalase activity decreased with Mo dose in the presence of AMF (from 105 to 87 mL O_2_ g^−1^ min^−1^) but reached its maximum at the intermediate dose in the absence of AMF (80 mL O_2_ g^−1^ min^−1^ at 0.3% Mo, compared with 56 and 65 mL O_2_ g^−1^ min^−1^ at 0% and 0.6% Mo). Dehydrogenase activity showed the most pronounced reversal: in non-AMF saline pots the highest activity occurred without Mo (20.1 µg TPF g^−1^ 24 h^−1^) and declined once Mo was added, whereas in AMF-inoculated saline pots the lowest activity occurred without Mo (15.5 µg TPF g^−1^ 24 h^−1^) and rose with Mo addition (to 18.3 µg TPF g^−1^ 24 h^−1^ at 0.3% Mo). Urease activity also differed between AMF and non-AMF pots, particularly under non-saline conditions, where it increased sharply with 0.3% Mo in the absence of AMF (40 µg N g^−1^ 24 h^−1^) but rose only modestly when AMF was present (26 µg N g^−1^ 24 h^−1^). These results indicate that, under the conditions tested, AMF and Mo interacted on soil biochemical activities rather than on plant biomass.

### 2.7. Correlation Structure Under Saline and Non-Saline Conditions

**Figure 7:**
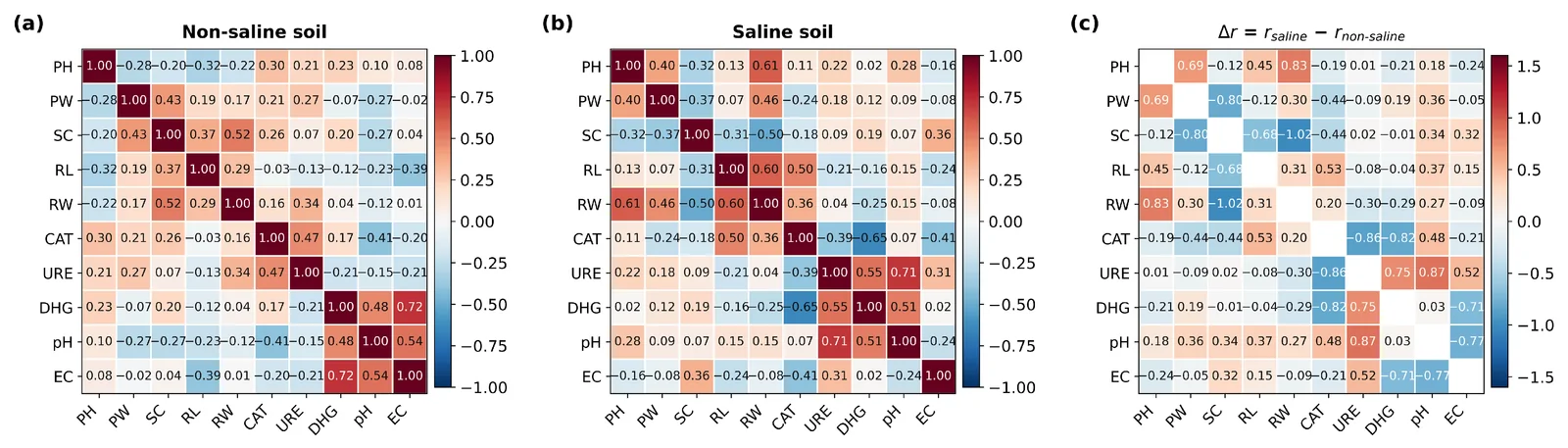
Pearson correlation matrices among the measured parameters in (**a**) non-saline and (**b**) saline soil, and (**c**) the difference between the two matrices (Δr = r_saline − r_non-saline; diagonal cells omitted). Cells are coloured from blue (negative) to red (positive) and numerical values are printed inside each cell. PH, plant height; PW, plant weight; SC, stomatal conductance; RL, root length; RW, root weight; CAT, catalase; URE, urease; DHG, dehydrogenase; pH, soil pH; EC, electrical conductivity.

Correlation matrices calculated separately for the two soil conditions differed in several respects (Figure 7). Under non-saline conditions, the strongest relationships were among soil chemical and biochemical parameters (dehydrogenase–pH, dehydrogenase–EC and pH–EC). Under saline conditions, root weight became correlated with several other parameters and stronger relationships appeared within the urease–dehydrogenase–pH group (urease–dehydrogenase changed from *r* = −0.21 to *r* = +0.55). Some associations changed direction between the two conditions: stomatal conductance and root weight shifted from *r* = +0.52 under non-saline conditions to *r* = −0.50 under salinity, and dehydrogenase and EC, which were positively correlated in non-saline soil (*r* = +0.72), were uncorrelated under salinity (*r* = +0.02). Given the number of replicates available, these differences are descriptive and should be interpreted as indicative rather than conclusive.

## 3. Discussion

### 3.1. Effects of Salinity on Plant and Soil Responses

Salinity was the dominant factor affecting both plant growth and soil biochemical processes in this experiment. It should be kept in mind, however, that the saline and non-saline soils also differed in carbonate content and pH (Section 3.6); the effects described here as effects of salinity are therefore more precisely the combined effect of salinity and these correlated soil properties, and the strongest causal wording should be read with that in mind. The reductions in plant weight, root length and root weight under saline conditions are consistent with previous reports that salinity suppresses plant growth through osmotic stress, ion toxicity and nutrient imbalance [3,4]. The increases in catalase and dehydrogenase activities are consistent with the activation of antioxidant defense and with continued microbial activity, whereas the decrease in urease activity points to a reduction in nitrogen-cycling enzymatic activity [3].

### 3.2. Stomatal Conductance Under Salinity

The 87% increase in stomatal conductance under salinity runs against the response most often described for glycophytes, in which salt-induced ABA accumulation closes stomata and lowers conductance [31], and it therefore deserves a closer look than a single sentence. Three features of this experiment are relevant. First, the stress applied was moderate rather than severe: the saline soil had an EC of 3.40 dS m^−1^ in the saturation extract, and soil moisture was held at 60–70% of field capacity throughout, so plants experienced an ion load without a superimposed water deficit. Stomatal closure under salinity is most pronounced when the osmotic component is strong or when soil water is limiting, and studies at moderate salinity have reported maintained or increased conductance in the same species that close their stomata at higher salt levels [2]. The meta-analysis of Chandrasekaran et al. [15] likewise found that gas-exchange responses to salinity vary considerably in direction and magnitude across experiments, so an increase at this level of stress is within the reported range rather than outside it.

Second, conductance was measured per unit leaf area on physiologically active, sunlit leaves between 11:00 and 15:00, whereas salinity reduced whole-plant biomass. Plants with less leaf area but a comparable evaporative demand can sustain a higher conductance per unit area while transpiring less in absolute terms, so a per-area increase does not necessarily imply higher whole-plant water use. The measurement is a snapshot at the end of the growth period and does not describe the trajectory of conductance over the season. Third, the two soils differed in more than salinity, and the saline soil was the more calcareous of the two, which affects nutrient availability and root growth independently of the salt load. Taken together, these considerations suggest that the increase reflects the specific combination of moderate salinity, maintained soil moisture and reduced leaf area in this pot experiment, rather than a general reversal of the stomatal response to salt. Confirming this would require leaf water potential, photosynthetic rate, transpiration and leaf Na^+^/K^+^ measurements alongside conductance, which were not part of the present measurement set and are listed among the planned additions in Section 3.6.

### 3.3. Role of AMF in Mitigating Stress and Modifying Soil–Plant Functions

AMF inoculation was associated with several changes that together indicate a regulatory rather than a purely growth-promoting role under saline conditions. AMF applied alone maintained root length and brought stomatal conductance back towards the non-saline range, and gave higher root fresh weight than the Mo-containing treatments under salinity. These observations are consistent with reports that AMF support water and nutrient uptake, modulate ion homeostasis and reduce oxidative damage under salinity [1,21,32]. It should be kept in mind that these treatment means were not significantly different (Table S3), so they indicate a direction worth following up rather than a demonstrated effect. The increase in catalase activity in AMF treatments under salinity is consistent with antioxidant activation, whereas the decrease in urease activity in the same treatments may reflect a reduced demand for soil nitrogen mineralization when AMF improves nitrogen acquisition through the symbiosis.

Dehydrogenase behaved in a way that is harder to place. Activity in AMF-inoculated saline pots was the lowest of all saline treatments, although still above every non-saline value. One reading is that photosynthate is partitioned preferentially towards the symbiotic partner under stress, leaving less labile carbon for free-living microorganisms [33], but the present dataset cannot separate this from at least three alternatives. Extraradical hyphae compete directly with free-living microorganisms for labile carbon and nutrients, so lower activity may reflect competition rather than altered plant partitioning [33]. Total dehydrogenase activity also depends on which organisms are present, and AMF are known to shift rhizosphere community composition [20], so a lower bulk value may correspond to a changed community rather than to reduced overall function. In addition, the TTC assay measures the reduction of an artificial electron acceptor and is sensitive to redox conditions and to competing oxidants in the soil solution [34], which is a particular consideration in the Mo-amended treatments. Distinguishing among these explanations requires ^13^C partitioning, microbial biomass carbon and community profiling, none of which were measured here; the interpretation above is therefore offered as one possibility among several rather than as a conclusion.

### 3.4. Molybdenum Effects and AMF × Mo Interactions

Molybdenum produced dose-dependent effects that differed in the presence and absence of AMF, and the magnitude of these differences varied with the parameter. For plant biomass, the AMF × Mo interaction was not statistically significant under saline conditions, and the responses to Mo with and without AMF followed comparable trajectories. For soil biochemical activities, in contrast, the interaction was highly significant for catalase, urease and dehydrogenase. It should be noted that the very large F-value obtained for dehydrogenase reflects the low within-treatment variability of this measurement in the present dataset, and the interaction for this parameter should therefore be interpreted primarily in terms of the direction of the response rather than its magnitude. The Mo doses applied here are well above the optimal range typically reported for legumes [24,30]. At these levels, the effect of Mo on soil enzymes appeared to be moderated by AMF: in AMF-containing treatments, catalase declined more gradually with Mo dose; dehydrogenase activity was higher in the AMF + Mo combinations than in AMF alone under salinity; and urease was strongly affected by Mo in non-AMF treatments but comparatively buffered when AMF was present. These results suggest that AMF modified the response of the soil biochemical compartment to Mo rather than additively reinforcing it.

### 3.5. Multivariate Structure and Comparison with Published Systems

The three multivariate analyses converge on the same picture, and the way they do so is informative in itself. In the PCA, PC1 alone accounted for 52% of the variance and separated the two soils completely, while the six treatments overlapped within each soil group. Hierarchical clustering produced the same split at the sample level and, at the variable level, divided the measured traits into a stress-associated cluster (stomatal conductance, catalase, dehydrogenase, EC) and a growth-associated cluster (plant height and weight, root length and weight, urease, pH). The correlation matrix reproduces this division as two blocks of positively inter-correlated variables that are negatively related to one another. In other words, the dataset is organized around a single dominant salinity axis, and the AMF and Mo treatments modulate positions along that axis rather than creating a second one.

This structure matches what has been reported for other salinity–AMF systems. Studies on soybean under saline–alkaline conditions [3] and on peanut in saline–alkaline soil [20] likewise found soil condition to be the primary source of variation, with inoculation effects appearing more clearly in soil biochemical and microbial variables than in biomass. The grouping of catalase, dehydrogenase and EC on the stress side is consistent with the review of microbial function in saline soils [17], which describes a shift towards oxidative and stress-related activity as salinity rises, and with reports that soil enzymes respond to salinity earlier and more strongly than plant growth traits [18,19]. The intermediate position of the mycorrhizal variables in the AMF-only ordination (Figure S1) is also in keeping with the meta-analytic finding that colonization and plant response to AMF are only loosely coupled under stress [15].

One point of contrast is worth noting. Several of the studies cited above report positive AMF effects on biomass, whereas biomass here was governed by soil condition alone. The most likely reasons are the short pot cycle, the restricted rooting volume, and the supra-optimal Mo background in four of the six treatments, all of which limit the extent to which a colonization benefit can be expressed as growth within a single season. The direction of the root-weight and stomatal responses to AMF alone is nevertheless the same as in those studies, which suggests that the difference is one of scale rather than of mechanism.

The correlation structure also changed between the two soils in ways that are biologically interpretable. The reversal of the stomatal conductance–root weight relationship, from *r* = +0.52 in non-saline soil to *r* = −0.50 under salinity, indicates that the coordination between root system size and stomatal behavior observed in unstressed plants did not persist under salt stress. Similarly, dehydrogenase activity and EC were positively related in non-saline soil (*r* = +0.72) but unrelated under salinity (*r* = +0.02), which is consistent with EC ceasing to act as a proxy for nutrient availability once it primarily reflects salt load. With three replicates per treatment combination, these comparisons remain descriptive, and they are reported as patterns to be tested rather than as established relationships.

### 3.6. Integrated Interpretation, Limitations and Agricultural Implications

Taken together, the results indicate that salinity was the dominant source of variation in the dataset; that AMF acted mainly on stomatal behavior, root biomass and the balance between antioxidant and microbial enzyme activities; and that Mo at the doses tested affected primarily soil biochemical activities, with a context-dependent interaction with AMF. For agricultural practice in salt-affected systems, these results support an approach in which AMF inoculation and Mo fertilization are considered jointly and calibrated together rather than applied as if their effects were simply additive. They also indicate that, where the objective is to sustain root biomass and keep stomatal behavior close to non-stressed values under salinity, AMF inoculation alone was the more promising option in this experiment.

Several constraints should be kept in view when reading these results. The two field soils differed in properties other than salinity: lime content was 26.6% in the saline soil against 11.0% in the non-saline soil, and organic matter was also somewhat higher (3.74% against 3.03%). Carbonate content affects phosphorus and micronutrient availability, the buffering of soil pH and the solubility of molybdate, and it can influence both AMF colonization and enzyme activity in its own right. The contrast between the two soils in this experiment is therefore best described as a contrast between a saline calcareous soil and a less calcareous non-saline soil, and the differences reported here as soil effects should be read as the combined effect of those properties rather than as the effect of salinity alone. The same applies to the macronutrient status of the two soils, which was not characterized in the present study. The internal comparisons that carry the main conclusions for AMF versus AMF + Mo within each soil and the AMF × Mo interaction terms are made within a single soil and are not affected by this limitation, but the between-soil comparisons are.

The Mo doses are a second, deliberate limitation. Both rates were far above agronomic practice and were chosen to characterize behavior under Mo excess. Therefore, they do not indicate what would happen at recommended rates, where the interaction with AMF could plausibly take a different direction [6,29].

The present study was conducted in pots with three replications per treatment combination, which limits the precision of the estimates and the generality of the conclusions. Future work should test these patterns under field conditions with larger replication, include Mo rates spanning the agronomic range (a gradient from a few to a few tens of mg Mo kg^−1^ soil) so that the shape of the response below the supra-optimal region can be resolved; use soils matched for carbonate content and macronutrient status or include these as covariates; characterize the inoculum and the soils more fully; and add measurements such as leaf K^+^/Na^+^ ratios, chlorophyll content, photosynthetic gas exchange, microbial biomass carbon and rhizosphere microbial community composition.

## 4. Materials and Methods

### 4.1. Experimental Site, Plant Material and Growing Conditions

The study was conducted under semi-controlled greenhouse conditions at the Research and Application Area of the Faculty of Agriculture, Department of Field Crops, Harran University. The experiment was established on 14 July 2023, and harvesting was carried out on 28 September 2023. Cowpea (*Vigna unguiculata* L.) cultivar “Karnıkara” was used as plant material. All plants were grown under uniform light and temperature conditions to minimize environmental variability. Soil moisture was maintained at 60–70% of field capacity so that plant responses reflected the studied factors (salinity, Mo and AMF) rather than water stress. Measurements were performed at the end of the growth period. Plastic pots (7.5 cm upper diameter, 4 cm base diameter, 13 cm height) were filled with 1 kg of soil each. Several seeds were sown per pot, and thinning was performed approximately 15 days after emergence, leaving one plant per pot. Soil was collected from two locations representing saline and non-saline conditions: Bozyazı village (Harran district, Şanlıurfa) and the Osmanbey Campus, both in Şanlıurfa Province, southeastern Türkiye. At each location, soil was taken from five points at 0–30 cm depth and combined into a homogeneous composite sample; sampling points were recorded by GPS. The physicochemical properties of the soils are given in Table 2.

### 4.2. Characterization and Background of the Experimental Soils

Both soils developed on the alluvial and colluvial deposits of the Harran Plain under a semi-arid Mediterranean climate and are fine-textured and calcareous. The soil series in the Bozyazı area is classified as a fine, smectitic, thermic Typic Torrert according to Soil Taxonomy and as a Chromic Vertisol according to the FAO/UNESCO system [35,36]. Selected properties of both soils are presented in Table 2. The EC, pH, CaCO_3_, and organic matter values represent the initial characteristics of the experimental soils, whereas nutrient values marked with an asterisk (*) are literature-based reference values for comparable soils of the Harran Plain [35,37,38].

The saline soil represents secondary, irrigation-induced salinization that developed in parts of the Harran Plain following the introduction of large-scale irrigation in the mid-1990s, driven by high evapotranspiration, limited drainage, and rising groundwater levels [39,40,41]. The saline soil was collected from a cultivated and irrigated area in Bozyazı, whereas the non-saline soil was collected from an uncultivated area of Harran University Osmanbey Campus that had not received irrigation or fertilizer inputs. Neither site had received Mo-containing fertilizers or mycorrhizal inoculum prior to sampling. However, the two soils also differed in properties other than salinity, most notably in lime content (26.62% and 10.96%).

### 4.3. Experimental Design and Treatments

The experiment was arranged as a 2 × 6 factorial in a completely randomized design with two soil types (saline and non-saline), six treatments and three replications. Molybdenum was applied as ammonium molybdate ((NH_4_)_6_Mo_7_O_24_·4H_2_O). Solutions prepared at 0.3% and 0.6% (*w*/*v*) were applied to the soil at a rate of 100 mL per pot at sowing, corresponding to 0.3 g and 0.6 g of ammonium molybdate per pot and to approximately 163 and 326 mg Mo kg^−1^ soil, respectively. Both rates are well above those recommended for legume production and were selected as supra-optimal exposures in order to characterize the behavior of the symbiosis under Mo excess, following the approach used in earlier AMF–Mo studies on maize and alfalfa [21,30]; they are not intended as fertilizer recommendations. All treatments were established in both saline and non-saline soils (Table 3).

* EC, pH, CaCO_3_ and organic matter values represent the initial characteristics of the soils used in the experiment. Texture and taxonomic information were obtained from published soil surveys for the corresponding locations. Nutrient values indicated with an asterisk (*) represent literature-based reference values for comparable soils of the Harran Plain and were not directly measured in the experimental soil samples but were taken from published surveys of comparable Harran Plain soils [35,37,38].

#### Mycorrhizal Inoculum

The AMF inoculum consisted of *Rhizophagus intraradices* (formerly *Glomus intraradices*), *Glomus constrictum* and *Glomus microcarpum*, and was applied at 10 g per pot (1% *w*/*w* of the potting soil) and mixed thoroughly into the soil before sowing. The inoculum was obtained from the laboratory culture collection of the Department of Soil Science and Plant Nutrition, Faculty of Agriculture, Harran University. The AMF culture had been propagated using maize (*Zea mays* L.) as a trap plant and consisted of spores, colonized root fragments, and extraradical hyphae. Non-inoculated treatments received no inoculum or corresponding substrate addition.

### 4.4. Measured Parameters

#### 4.4.1. Plant Measurements

Plant height and root length were measured in centimeters and fresh plant and root weights in grams. Stomatal conductance was measured with a Decagon SC-1 leaf porometer (Decagon Devices, Pullman, WA, USA) between 11:00 and 15:00 on physiologically active, sunlit leaves [42]. All readings were taken from leaves at the same position and comparable physiological stage to minimize measurement error.

#### 4.4.2. Soil Chemical and Biochemical Analyses

All analyses were carried out on air-dried soil passed through a 2 mm sieve. Soil pH was determined in a 1:2.5 soil–water suspension with a pH meter, and electrical conductivity (EC) was measured in the same suspension with a conductivity meter and expressed in µS cm^−1^ [43]. Catalase activity was determined volumetrically following Beck [44]: a moist soil sample was reacted with hydrogen peroxide in a closed vessel, and the volume of oxygen released was recorded manometrically. Activity is expressed as mL O_2_ g^−1^ dry soil min^−1^, obtained by dividing the total oxygen volume released by the sample mass and by the incubation time; the incubation was carried out at 20 °C for 3 min using 5 g of soil, 10 mL of phosphate buffer (pH 7.0), and 5 mL of 3% H_2_O_2_. Because the assay is volumetric and uses excess substrate, the values obtained are considerably higher than those reported for colorimetric permanganate-titration variants of the method, and the two are not directly comparable. Urease activity was determined spectrophotometrically by measuring the ammonia produced from urea hydrolysis [45] and is expressed as µg N g^−1^ dry soil 24 h^−1^. Dehydrogenase activity was determined by the TTC (2,3,5-triphenyl tetrazolium chloride) method; the triphenyl formazan (TPF) formed after incubation was measured at 485 nm and expressed as µg TPF g^−1^ dry soil 24 h^−1^ [34].

#### 4.4.3. Mycorrhizal Analyses

AMF spore density in soil and root colonization were evaluated as described by Boyno et al. [46]. Soil samples were subjected to the appropriate suspension procedure, and spores were counted under a stereomicroscope at 40× magnification. The total number of AMF spores in 1 g of rhizosphere soil was calculated as TSN = SN × W/S, where TSN is the total spore number per gram of rhizosphere soil, SN is the number of spores in 1 mL of suspension, W is the volume of water used (mL) and S is the amount of soil used (g). The root mycorrhizal colonization rate (%M) and mycorrhizal frequency (%F) were determined by the semi-quantitative method based on the 0–5 scale of Trouvelot et al. [47]. Roots were cleared in KOH and stained with trypan blue before assessment, and 9 root segments per replicate were scored. AMF spore density was determined by counting the spores under a binocular stereomicroscope (Leica, Hamburg, Germany) at 8×–12× magnification.

### 4.5. Statistical Analysis

#### 4.5.1. ANOVA and Univariate Statistics

Data were analyzed by two-way analysis of variance (ANOVA) to evaluate the effects of soil type, treatment and their interaction, using JMP Pro 17 (SAS Institute Inc., Cary, NC, USA). Normality of residuals was assessed with the Shapiro–Wilk test and homogeneity of variances with Levene’s test. Tukey’s HSD test (α = 0.05) was used for mean separation.

#### 4.5.2. Multivariate Analysis

All variables were z-score standardized before multivariate analysis. Principal component analysis (PCA) was applied to summarize the structure of variation in the dataset. Hierarchical clustering was performed using Euclidean distance and Ward’s linkage, and the results were visualized as a heatmap. Linear relationships between variables were assessed by Pearson correlation analysis, both across the whole dataset and separately for saline and non-saline samples.

#### 4.5.3. Mo × AMF Dose–Response Analysis

The Mo × AMF interaction was examined by plotting parameter means against Mo dose (0, 0.3 and 0.6%) separately for plants with and without AMF inoculation, with standard errors of the mean. The interaction was tested formally by a two-way ANOVA with Mo dose (categorical) and AMF status (with/without) as factors, and applied to data from saline pots, and the F- and *p*-values of the interaction term were reported. The dose–response analysis was performed in Python 3.11 (pandas, numpy, scipy, statsmodels and matplotlib).

## 5. Conclusions

This study examined the effects of AMF inoculation and two molybdenum doses on cowpea grown under saline and non-saline conditions. Salinity reduced biomass and root growth, increased stomatal conductance and the activities of catalase and dehydrogenase, and decreased urease activity. None of the treatments changed the plant growth traits significantly, and the treatment × soil interaction was not significant for any of them, so plant growth in this experiment responded to soil condition alone. AMF applied alone was the treatment most closely associated with maintained root fresh weight and near-control stomatal conductance under salinity, and it produced the highest mycorrhizal colonization. Molybdenum, at the supra-optimal doses tested, did not enhance AMF performance under saline conditions; on the contrary, adding Mo to AMF reduced mycorrhizal density and colonization. The AMF × Mo interaction was statistically significant for soil enzyme activities but not for plant biomass. These results indicate that AMF and Mo act on largely separable components of the cowpea plant–soil system, and that integrated management strategies in salt-affected agroecosystems should consider the two inputs jointly and calibrate them with attention to dose and to the specific parameters of interest. Because the two soils differed in carbonate content as well as in salinity, and because both Mo rates lie above agronomic practice, field-scale validation using soils matched for carbonate content and a Mo gradient within the recommended range is needed before these observations can be translated into practice.

## Figures and Tables

**Table 1:** AMF × Mo interaction terms from a two-way ANOVA on data from saline pots only, with Mo dose (categorical: 0, 0.3, 0.6%) and AMF status (with/without) as factors.

Parameter	F	*p*
Plant fresh weight	0.08	0.920
Root fresh weight	1.09	0.368
Stomatal conductance	0.95	0.415
Catalase	43.01	<0.001
Urease	284.92	<0.001
Dehydrogenase	5076.99	<0.001

**Table 2:** Physicochemical properties of the soils used in the experiment. Electrical conductivity (EC) was determined in a saturation extract.

Property	Saline Soil	Non-Saline Soil
EC (dS m^−1^)	3.40	0.88
pH	7.20	7.82
Lime, CaCO_3_ (%)	26.62	10.96
Organic matter (%)	3.74	3.03
Texture class	Clay	Clay
Total N (%) *	0.29	0.38
Available P (mg kg^−1^) *	4.00	4.23
Exchangeable K (mg kg^−1^) *	≈180	175.22
Soil classification *	Typic Torrert/Chromic Vertisol	Typic Torrert/Chromic Vertisol
Salinity origin *	Secondary (irrigation-induced)	Not saline

(*) nutrient values are literature-based reference values for comparable Harran Plain soils (not measured directly in the experimental soils).

**Table 3:** Treatments applied under saline and non-saline conditions.

Treatment Code	Non-Saline Soil	Saline Soil
T1	Control	Control
T2	0.3% Mo	0.3% Mo
T3	0.6% Mo	0.6% Mo
T4	AMF	AMF
T5	AMF + 0.3% Mo	AMF + 0.3% Mo
T6	AMF + 0.6% Mo	AMF + 0.6% Mo

## Data Availability

The data presented in this study are available from the corresponding author on reasonable request.

## Supplementary Materials

The following supporting information can be downloaded at: https://www.mdpi.com/article/10.3390/plants15182816/s1, Table S1: Two-way analysis of variance (ANOVA) for the effects of treatment, soil type and their interaction on the plant growth, physiological and soil parameters of cowpea; Table S2: Two-way ANOVA for the effects of treatment, soil type and their interaction on the mycorrhizal parameters, analysed across the three AMF-containing treatments (AMF, AMF + 0.3% Mo, AMF + 0.6% Mo); Table S3: Descriptive statistics (mean ± standard error, n = 3) and Tukey’s HSD mean separation for each parameter under the six treatments in non-saline and saline soil; Figure S1: Principal component analysis restricted to the AMF-inoculated pots and including the mycorrhizal parameters (mycorrhizal density, mycorrhizal frequency and spore number).

## Abbreviations

Abbreviation	Definition
AMF	Arbuscular mycorrhizal fungi
ANOVA	Analysis of variance
CAT	Catalase
DHG	Dehydrogenase
EC	Electrical conductivity
Mo	Molybdenum
PCA	Principal component analysis
ROS	Reactive oxygen species
SC	Stomatal conductance
TPF	Triphenyl formazan
TTC	2,3,5-Triphenyl tetrazolium chloride
%F	Mycorrhizal frequency
%M	Mycorrhizal density
URE	Urease
HSD	Honestly significant difference
