# Supplementary material for: Arbuscular Mycorrhizal Fungi and Molybdenum Act on Different Components of the Cowpea Plant–Soil System Under Salinity
Source: Plants (Basel). 2026 Sep 14;15(18):2816. doi: 10.3390/plants15182816 (PMC13610599; doi:10.3390/plants15182816)
Supplement: Supplementary file 1 [file plants-15-02816-s001.zip › plants-4478227-supplementary.pdf]

Supplementary Materials

Arbuscular Mycorrhizal Fungi and Molybdenum Act on Different Components of the Cowpea Plant–Soil System under Salinity

Table S1. Two-way analysis of variance (ANOVA) for the effects of treatment, soil type and their interaction on the plant growth, physiological and soil parameters of cowpea. F-values are given with their significance; degrees of freedom were 5 (treatment), 1 (soil), 5 (treatment × soil) and 24 (error).

Parameter	Treatment (T)		Soil (S)		T × S	
	F	***p***	***F***	***p***	***F***	***p***
Plant height (cm plant^{-1})	1.57	0.208 ns	0.31	0.585 ns	2.25	0.081 ns
Plant fresh weight (g plant^{-1})	0.97	0.454 ns	16.48	<0.001 ***	1.42	0.252 ns
Stomatal conductance (mmol m^{-2} s^{-1})	1.50	0.228 ns	22.00	<0.001 ***	0.90	0.499 ns
Root length (cm plant^{-1})	1.35	0.279 ns	19.18	<0.001 ***	1.74	0.163 ns
Root fresh weight (g plant^{-1})	0.88	0.507 ns	51.57	<0.001 ***	1.51	0.223 ns
Catalase (mL O_2 g^{-1} min^{-1})	69.09	<0.001 ***	4536.77	<0.001 ***	65.28	<0.001 ***
Urease (µg N g^{-1} 24 h^{-1})	299.70	<0.001 ***	699.56	<0.001 ***	123.97	<0.001 ***
Dehydrogenase (µg TPF g^{-1} 24 h^{-1})	1992.88	<0.001 ***	64145.16	<0.001 ***	1755.78	<0.001 ***
Soil pH	39.39	<0.001 ***	543.49	<0.001 ***	15.92	<0.001 ***
EC (µS cm^{-1})	6992.83	<0.001 ***	47992.70	<0.001 ***	3557.74	<0.001 ***

Table S2. Two-way ANOVA for the effects of treatment, soil type and their interaction on the mycorrhizal parameters, analysed across the three AMF-containing treatments (AMF, AMF + 0.3% Mo, AMF + 0.6% Mo). Degrees of freedom were 2 (treatment), 1 (soil), 2 (treatment × soil) and 12 (error).

Parameter	Treatment (T)		Soil (S)		T × S	
	F	***p***	***F***	***p***	***F***	***p***
Spore number (g^{-1} soil)	4.76	0.030 *	20.89	<0.001 ***	2.37	0.136 ns
Mycorrhizal frequency (%F)	8.33	0.005 **	21.33	<0.001 ***	34.33	<0.001 ***
Mycorrhizal density (%M)	3.85	0.051 ns	31.76	<0.001 ***	85.04	<0.001 ***

Table S3. Descriptive statistics (mean ± standard error, n = 3) and Tukey's HSD mean separation for each parameter under the six treatments in non-saline and saline soil. Within a parameter, means followed by the same letter are not significantly different at $p < 0.05$; mean separation compares the twelve treatment × soil combinations. Mycorrhizal parameters were assessed only in the three AMF-containing treatments.

Soil	Treatment	Plant height (cm $plant^{-1}$)	Plant fresh weight (g $plant^{-1}$)	Stomatal conductance (mmol m^{-2} s^{-1})	Root length (cm $plant^{-1}$)	Root fresh weight (g $plant^{-1}$)	Catalase (mL O_2 g^{-1} min^{-1})	Urease (µg N g^{-1} 24 h^{-1})	Dehydrogenase (µg TPF g^{-1} 24 h^{-1})	Soil pH	EC (µS cm^{-1})	Spore number (g^{-1} soil)	Mycorrhizal frequency (%F)	Mycorrhizal density (%M)
Non-saline	Control	19.5±2.2 a	3.26±0.63 a	91.2±19.1 a	40.4±7.6 a	1.64±0.20 a	15.0±0.6 g	20.6±0.2 c	13.6±0.0 g	7.57±0.00 b	187±1 h	—	—	—
Non-saline	0.3% Mo	19.1±2.0 a	3.16±0.32 a	78.3±11.0 a	31.3±3.7 a	2.02±0.40 a	19.0±0.6 g	40.0±0.1 a	13.4±0.0 h	7.57±0.01 a	170±1 i	—	—	—
Non-saline	0.6% Mo	17.0±1.5 a	3.04±0.04 a	78.9±22.4 a	31.6±3.2 a	1.69±0.19 a	15.0±0.6 g	17.0±0.1 d	13.4±0.0 h	7.54±0.01 b	209±1 g	—	—	—
Non-saline	AMF	17.7±1.0 a	2.51±0.29 a	69.4±3.4 a	34.2±3.3 a	1.70±0.08 a	11.0±0.6 g	13.1±0.5 e	13.6±0.0 g	7.60±0.01 a	214±1 g	193±18 b	51.9±3.7 c	0.51±0.04 c
Non-saline	AMF+0.3% Mo	20.3±2.0 a	3.09±0.21 a	92.7±5.3 a	31.1±3.2 a	1.85±0.20 a	29.0±1.7 f	25.7±1.2 b	13.7±0.0 f	7.56±0.01 b	254±1 f	153±7 b	81.5±3.7 a	1.77±0.17 b
Non-saline	AMF+0.6% Mo	19.1±0.5 a	3.03±0.22 a	83.0±20.7 a	26.2±4.0 a	1.80±0.12 a	12.0±1.2 g	20.7±0.1 c	13.8±0.0 f	7.61±0.00 a	457±1 a	200±23 b	55.6±0.0 b	2.11±0.06 b
Saline	Control	21.2±1.3 a	2.86±0.08 a	139±22 a	20.9±3.8 a	0.85±0.08 b	56.0±2.3 e	21.9±0.1 c	20.1±0.1 a	7.52±0.01 c	381±1 d	—	—	—
Saline	0.3% Mo	16.8±1.3 a	2.16±0.30 a	159±45 a	17.1±4.3 b	0.64±0.27 b	80.0±1.2 c	19.2±0.4 c	16.8±0.0 d	7.50±0.01 c	379±1 d	—	—	—
Saline	0.6% Mo	16.6±1.0 a	2.25±0.06 a	168±49 a	17.7±3.3 b	0.56±0.06 b	65.0±2.9 d	7.88±0.49 f	16.7±0.0 d	7.39±0.01 e	410±1 c	—	—	—
Saline	AMF	21.9±1.6 a	2.65±0.35 a	81.7±8.5 a	29.4±3.8 a	1.38±0.49 a	105±1 a	9.27±0.15 f	15.5±0.0 e	7.46±0.01 d	352±1 e	300±23 a	77.8±0.0 a	3.66±0.17 a
Saline	AMF+0.3% Mo	15.3±1.8 a	1.79±0.47 a	181±42 a	25.5±0.4 a	0.54±0.12 b	91.0±2.9 b	9.76±0.23 f	18.3±0.0 b	7.49±0.01 c	354±1 e	227±18 a	66.7±0.0 b	1.55±0.26 b
Saline	AMF+0.6% Mo	18.1±0.8 a	2.09±0.06 a	193±13 a	25.7±1.3 a	1.02±0.02 a	87.0±1.7 b	18.9±1.1 d	17.2±0.0 c	7.50±0.01 c	429±1 b	227±18 a	74.1±3.7 a	1.40±0.16 b

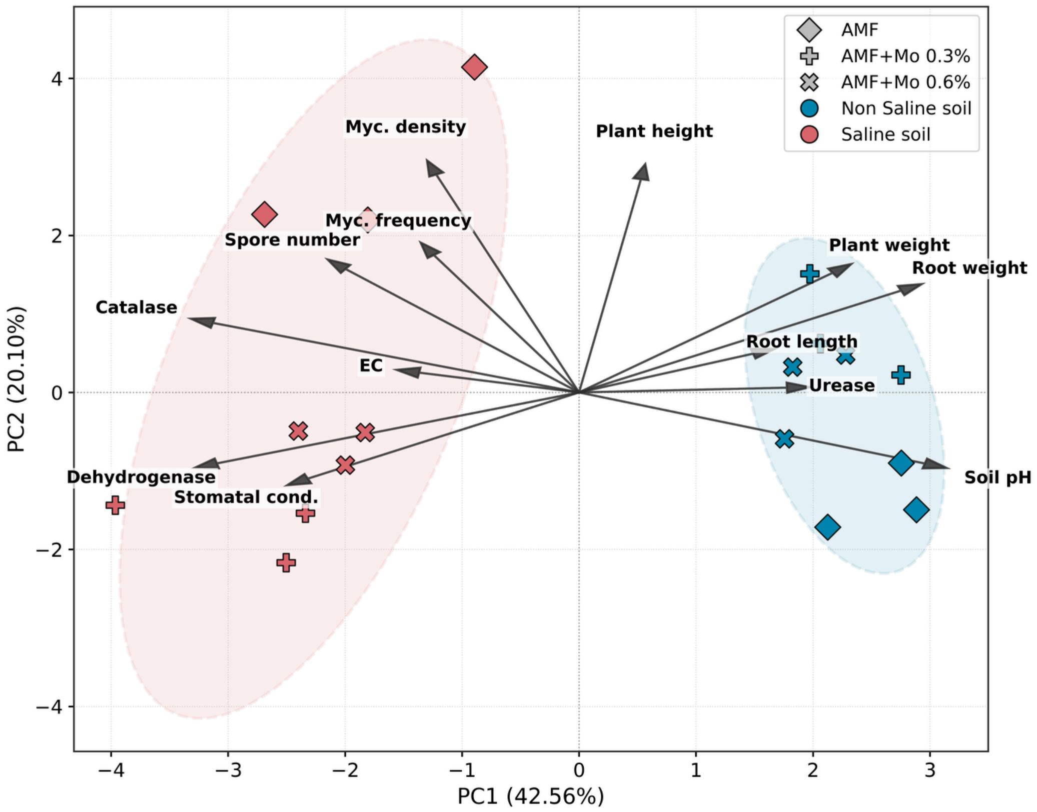

Figure S1. Principal component analysis restricted to the AMF-inoculated pots and including the mycorrhizal parameters (mycorrhizal density, mycorrhizal frequency and spore number). PC1 explained 42.56% and PC2 20.10% of the total variance.
